# Topological Heat Transport and Symmetry-Protected Boson Currents

**DOI:** 10.1038/s41598-017-06722-x

**Published:** 2017-07-25

**Authors:** Ángel Rivas, Miguel A. Martin-Delgado

**Affiliations:** 1 Departamento de Física Teórica I, Universidad Complutense, 28040 Madrid, Spain; 2 CCS -Center for Computational Simulation, Campus de Montegancedo UPM, 28660 Boadilla del Monte, Madrid, Spain

## Abstract

The study of non-equilibrium properties in topological systems is of practical and fundamental importance. Here, we analyze the stationary properties of a two-dimensional bosonic Hofstadter lattice coupled to two thermal baths in the quantum open-system formalism. Novel phenomena appear like chiral edge heat currents that are the out-of-equilibrium counterparts of the zero-temperature edge currents. They support a new concept of dissipative symmetry-protection, where a set of discrete symmetries protects topological heat currents, differing from the symmetry-protection devised in closed systems and zero-temperature. Remarkably, one of these currents flows opposite to the decreasing external temperature gradient. As the starting point, we consider the case of a single external reservoir already showing prominent results like thermal erasure effects and topological thermal currents. Our results are experimentally accessible with platforms like photonics systems and optical lattices.

## Introduction

Topological insulators represent a new state of matter that have attracted much attention due to their exotic physical properties and its potential applications in spintronics, photonics, etc., that may revolutionize these fields^1–4^.

One of the most active areas in topological insulators is their quantum simulation with the goal of realizing novel physical properties that are otherwise very difficult to realize in a standard condensed matter system^5–7^. Among these quantum simulators, bosonic systems such as ultracold atoms in optical lattices or photonic chips, stand up as versatile and promising experimental platforms that have achieved enormous progress and points towards near-future technological applications^8–15^.

Whereas these systems have been extensively studied for the idealized and isolated case, very little is known about the response of these setups to the action of external thermal fluctuations or external dissipation^16–21^. This is both true when the system is perturbed by some external heat bath or in an out-of-equilibrium situation where the system interacts with two different heat sources. Then, a natural question that arises is to what extent the topological properties of these systems affect heat currents and transport under these circumstances. In particular, and very importantly, are there new topological heat currents showing exotic behavior?

In this work we have addressed these novel and relevant issues showing that the exposition to thermal sources has not necessarily a detrimental effect. It can actually produce new topological features outside the paradigm of closed systems, that may have also technological applications. Among these results we may highlight:

(i) Thermal Erasure Effect (TEE): There exists a wide range of temperatures such that the current becomes more localized on the edge than for an individually excited edge mode. In particular, bulk currents are negligible in comparison to the edge ones as an effect of thermal fluctuations.
(ii) Topological Thermal Currents (TTC): The edge currents driven in the system by the presence of a single thermal bath are topologically protected against disorder.
(iii) Edge Nonequilibrium Crosscurrent (ENC): When the system is in contact with two baths at different temperatures (i.e. nonthermal equilibrium situation), a chiral current is also induced so that on one edge of the system the current flows in opposite direction to the heat. This can be thought of as a local entropy decrease on that edge^22^. Thus, the violation of the second law (Clausius form) by the nonequilibrium crosscurrent is just apparent since the net heat flow is from the hottest bath to the coldest one, as it should be.
(iv) Symmetry-Protected Non-Equilibirum Currents (SPNC). As a difference with standard topological currents, the nonthermal equilibrium currents present selective robustness. The edge current is immune to the presence of disorder provided that it satisfies a particular global spatial symmetry.

This notion of SPNC is motivated by a similar notion for topological insulators environmentally isolated (closed systems), but there is one important difference. Namely, the currents here are effectively protected by discrete symmetries as clearly shown in our numerical simulations for a wide range of temperatures. However, at very high temperatures the protection ceases to be operative, as it may be expected. This has mathematical implications, e.g. it is not possible to ascribe a usual Chern number (integer) to these currents as in the standard closed case. Nevertheless, this comprises the main novel feature of our study, the concept of *dissipative symmetry*-*protection*: an effective notion of symmetry-protected heat currents valid for open systems. Moreover, the SPNC are switchable without altering the thermal baths: by controlling the orientation of the external magnetic field its chirality can be modified at will (see Fig. S2 in the supplementary information document). This may represent a new way to technologically exploit heat flows.

## Results

### System and Dynamics

**Figure 1:**
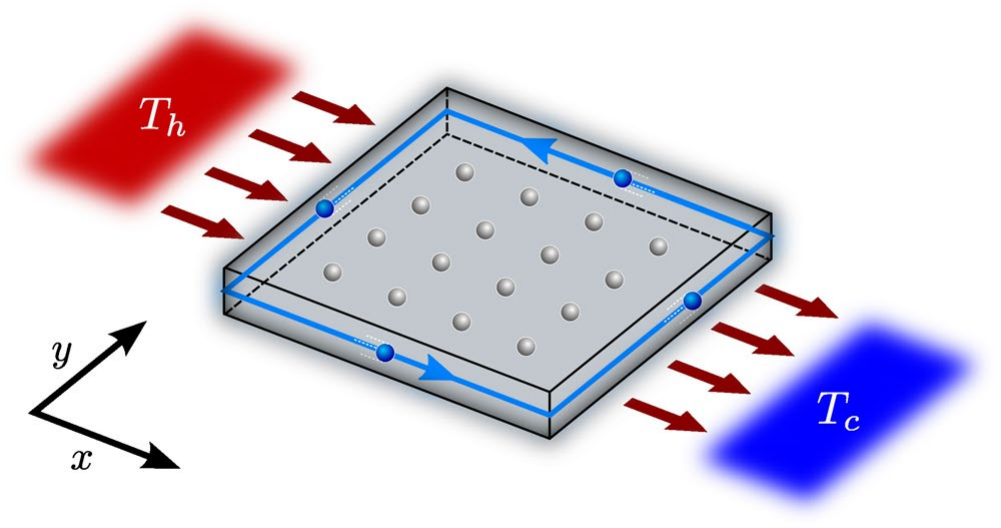
Schematic arrangement considered throughout this work. A Hofstadter boson system is set in contact with two oppositely sited thermal baths at respective temperatures *T*_*h*_ (hot bath) and *T*_*c*_ (cold bath). As a result, exotic chiral currents are induced on the system. The brown arrows show the direction of heat.

We consider a *N* × *N* square lattice of bosonic modes in contact with two sets of local (bosonic) thermal reservoirs, one, on the left, at temperature *T*_*h*_ and other on the right, at temperature *T*_*c*_ (without lost of generality we shall assume *T*_*h*_ ≥ *T*_*c*_) as depicted in Fig. 1. The lattice Hamiltonian is assumed to be

1 $${H}_{S}=\sum _{x,y}\hslash {\omega }_{0}{a}_{x,y}^{\dagger }{a}_{x,y}+V,$$

with

2 $$V=-\hslash J\sum _{x,y}{a}_{x+\mathrm{1,}y}^{\dagger }{a}_{x,y}{e}^{-2\pi \alpha iy}+{a}_{x,y+1}^{\dagger }{a}_{x,y}+{\rm{h}}{\rm{.c}}{\rm{.}}$$

This is the bosonic version of the Hofstadter model for the integer quantum Hall effect^23^. This bosonic Hofstadter Hamiltonian has been obtained in controlled systems like ultracold gases in optical lattices subject to laser induced tunneling^13–15^, and photonic circuits arranging differential optical paths^11^. Furthermore, similar dynamics can also be found in photonic crystals^10, 24^. These systems reproduce the effect of the magnetic flux *α* by several artificial techniques allowing for the exploration of quantum Hall physics with neutral atoms or photons. The interaction with local reservoirs is modeled by a sum of individual Hamiltonians accounting for a standard quadratic interaction:

3 $${H}_{SR}=\sum _{j,y}{g}_{j}({A}_{j,y}+{A}_{j,y}^{\dagger })({a}_{\mathrm{1,}y}+{a}_{\mathrm{1,}y}^{\dagger })+{g}_{j}({B}_{j,y}+{B}_{j,y}^{\dagger })({a}_{N,y}+{a}_{N,y}^{\dagger }).$$

Here, *A*_*j*, *y*_ and *B*_*j*, *y*_ denote bosonic operators of the mode with frequency *ω*_*j*_ of the reservoir at position *y*, at the left and right hand side (i.e. hot and cold), respectively; and *g*_*j*_ is the coupling constant assumed to be the same for all reservoirs. Although the assumption of local reservoirs is quite natural in the context of individual addressing, where access to each individual site of the lattice is possible, in the practice it turns out to be reasonably valid for a wide variety of physical situations.

Assuming weak system-bath couplings and following the usual steps for the derivation of the master equation (see Methods), we obtain

4 $$\begin{array}{rcl}\frac{d\rho }{dt} & = & \gamma (\rho )=-\frac{i}{\hslash }[{H}_{S},\rho ]+{\sum }_{k}\gamma \{{s}_{k}[{\bar{n}}_{k}({T}_{h})+\mathrm{1]}+{r}_{k}[{\bar{n}}_{k}({T}_{c})+\mathrm{1]}\}({b}_{k}\rho {b}_{k}^{\dagger }-\frac{1}{2}\{{b}_{k}^{\dagger }{b}_{k},\rho \})\\  &  & \quad \quad \,\quad +{\sum }_{k}\gamma [{s}_{k}{\bar{n}}_{k}({T}_{h})+{r}_{k}{\bar{n}}_{k}({T}_{c})]({b}_{k}^{\dagger }\rho {b}_{k}-\frac{1}{2}\{{b}_{k}{b}_{k}^{\dagger },\rho \}),\end{array}$$

where ${\mathcal L}$ represents the Liouvillian operator, *b*_*k*_ stands for the normal modes of *H*_*S*_, ${\bar{n}}_{k}(T)={\{\exp [\hslash {\omega }_{k}/({k}_{B}T)]-\mathrm{1\}}}^{-1}$ denotes the mean number of bosons with frequency *ω*_*k*_ and temperature *T*, and *γ* is a constant that depends on the strength of the coupling $\gamma \sim {g}_{j}^{2}$. Furthermore the constant*s s*_*k*_ and *r*_*k*_ are related to the coordinates in real space of “one-particle” eigenfunctions *ψ*_*k*_(*x*, *y*) of *H*_*S*_, via

5 $${s}_{k}=\sum _{y=1}^{N}{|{\psi }_{k}\mathrm{(1,}y)|}^{2}\quad {\rm{and}}\quad {r}_{k}=\sum _{y=1}^{N}{|{\psi }_{k}(N,y)|}^{2}.$$

They correspond to left (*x* = 1) and right (*x* = *N*) sides near the reservoirs, respectively.

We aim at studying the heat current in the asymptotic limit, once the system has reached stability. For the equilibrium situation *T*_*h*_ = *T*_*c*_ = *T*, the decay rate becomes *γ*(*s*_*k*_ +  *r*_*k*_) and the master equation (4) drives the system towards thermal equilibrium with the baths, so that the steady state is ${\rho }_{{\rm{ss}}}:={\mathrm{lim}}_{t\to \infty }\rho (t)={\rho }_{\beta }=\frac{{e}^{-\beta {H}_{S}}}{Z}$ with *β* = 1/(*k*_*B*_ *T*) and *Z* = Tr[exp(−*βH*_*S*_)]. In the general nonthermal equilibrium case *T*_*h*_ > *T*_*c*_, the steady state can be written as ${\rho }_{{\rm{s}}{\rm{s}}}={W}^{-1}\,\exp (-{\sum }_{k}\frac{\hslash {\omega }_{k}}{{k}_{B}{T}_{k}^{{\rm{e}}ff}}{b}_{k}^{\dagger }{b}_{k})$ where *W* is a normalization constant and the quantity ${T}_{k}^{{\rm{eff}}}$ plays the role of a mode-dependent effective temperature with the form

6 $${T}_{k}^{{\rm{e}}{\rm{f}}{\rm{f}}}:=\frac{\hslash {\omega }_{k}}{{k}_{B}\,{\rm{l}}{\rm{o}}{\rm{g}}\{\frac{\exp ({\textstyle \tfrac{\hslash {\omega }_{k}}{{k}_{B}{T}_{h}}})[\exp ({\textstyle \tfrac{\hslash {\omega }_{k}}{{k}_{B}{T}_{c}}})-1]\,{s}_{k}+\exp ({\textstyle \tfrac{\hslash {\omega }_{k}}{{k}_{B}{T}_{c}}})[\exp ({\textstyle \tfrac{\hslash {\omega }_{k}}{{k}_{B}{T}_{h}}})-1]{r}_{k}}{[\exp ({\textstyle \tfrac{\hslash {\omega }_{k}}{{k}_{B}{T}_{c}}})-1]\,{s}_{k}+[\exp ({\textstyle \tfrac{\hslash {\omega }_{k}}{{k}_{B}{T}_{h}}})-1]{r}_{k}}\}}.$$

Since the lattice holds a ΘΣ_*y*_ symmetry [Θ: time-reversal (changing *α* ↔ −*α*), Σ_*y*_: 2D reflection across the *y* axis], we have |*ψ*_*k*_(1, *y*)|^2^ = |*ψ*_*k*_(*N*, *y*)|^2^ and therefore

7 $${s}_{k}={r}_{k}\mathrm{.}$$

As a consequence, in general terms, the physical properties described by the master equation (4) are invariant under all symmetries respecting both *H*_*S*_ and Eq. (7), and thus the Liouvillian operator ${\mathcal L}$. These are ΘΣ_*y*_ and *R*_*π*_, a *π*-rotation along the orthogonal direction to the lattice [*R*_*π*_ *ψ*_*k*_(1, *y*) =* ψ*_*k*_(*N*, *N* + 1 − *y*)]. As commented, for *T*_*h*_ = *T*_*c*_ = *T*, the physics in the stationary limit is independent of *s*_*k*_ and *r*_*k*_ because their effect on the master equation (4) is just a renormalization of *γ* →* γ*(*s*_*k*_ + *r*_*k*_), so that the symmetries of *r*_*k*_ and *s*_*k*_ do not play any significant role. This is consequent with the fact that no original spatial symmetry is broken as no temperature gradient is applied. However, things are different if *T*_*h*_ > *T*_*c*_. Specifically, we can await for robustness of the chiral currents in the Hofstadter model also in the nonthermal equilibrium situation, as least if the spatial distribution of defects remains invariant under ΘΣ_*y*_ or *R*_*π*_ such that Eq. (7) is satisfied. If the latter is not the case, the physical properties of the master equation (4) may be affected and there is no guarantee that the robustness of the currents was preserved. Hence, a distribution of defects that changes by ΘΣ_*y*_ and *R*_*π*_, might destabilize the chiral current. Mathematically, this corresponds to a pair of ${{\mathbb{Z}}}_{2}$ symmetries, ${{\mathbb{Z}}}_{2}\equiv \{{\mathbb{1}},{R}_{\pi }\}$ and ${{\mathbb{Z}}}_{2}^{\ast }\equiv \{{\mathbb{1}},{\rm{\Theta }}{{\rm{\Sigma }}}_{{\rm{y}}}\}$. Note that different symmetry-protected topological behavior may be expected from the fact that the system cannot be deformed from the thermal to nonthermal situations in a continuous way avoiding symmetry-breaking.

### Internal and External Currents

We shall distinguish between two types of currents, the external ones, which describe the exchange of energy between system and baths, and the internal ones, that concern to the transport inside the *N* × *N* lattice system array. The external currents operators are derived from the master equation (4) and the continuity equation for the total energy (see Methods), and take the form

8 $${{\mathcal{J}}}_{h}\,:=-\hslash \sum _{k}{\omega }_{k}{\gamma }_{k}{s}_{k}[{b}_{k}^{\dagger }{b}_{k}-{\bar{n}}_{k}({T}_{h})],$$

9 $${{\mathcal{J}}}_{c}\,:=-\hslash \sum _{k}{\omega }_{k}{\gamma }_{k}{r}_{k}[{b}_{k}^{\dagger }{b}_{k}-{\bar{n}}_{k}({T}_{c})],$$

for the exchange with hot and cold bath, respectively. In the steady state limit, their expectation values become

10 $${\langle {{\mathcal{J}}}_{h,c}\rangle }_{{\rm{s}}{\rm{s}}}=\hslash \sum _{k}{\omega }_{k}{\gamma }_{k}{r}_{k}{s}_{k}[\frac{{\bar{n}}_{k}({T}_{h,c})-{\bar{n}}_{k}({T}_{c,h})}{{s}_{k}+{r}_{k}}].$$

Since *T*_*h*_ ≥ *T*_*c*_, ${\langle {{\mathcal{J}}}_{h}\rangle }_{{\rm{s}}{\rm{s}}}=-{\langle {{\mathcal{J}}}_{c}\rangle }_{{\rm{s}}{\rm{s}}}\ge 0$ and the heat current abides with the second law flowing from the hot bath to the system and from system to the cold bath.

External currents are less exposed to topological properties (e.g. their values are quite independent of the value of *α*) than internal currents. This is due to the fact that the system-bath coupling *H*_*SR*_ does not enjoy any special topological feature. These are present in the intersystem coupling *V*, Eq. (2), which is directly related to internal currents.

Gauge invariant operators for internal currents are derived in similar fashion from the continuity equation for site populations $\langle {a}_{x,y}^{\dagger }{a}_{x,y}\rangle$ in terms of the total Hamiltonian *H*_*S*_ + *H*_*SR*_, they read

11 $${{\mathcal{J}}}_{(\to x),y}\,:=iJ({a}_{x,y}^{\dagger }{a}_{x-\mathrm{1,}y}{e}^{-2\pi \alpha iy}-{a}_{x-\mathrm{1,}y}^{\dagger }{a}_{x,y}{e}^{2\pi \alpha iy}),$$

12 $${{\mathcal{J}}}_{x,(\to y)}\,:=iJ({a}_{x,y}^{\dagger }{a}_{x,y-1}-{a}_{x,y-1}^{\dagger }{a}_{x,y}),$$

where the subindex (→*x*) is a short notation for (*x* − 1 → *x*). Thus ${{\mathcal{J}}}_{(\to x),y}$ denotes the operator for the current leaving site (*x* − 1, *y*) and entering in (*x*, *y*). Similarly for (→*y*). Note that these internal currents describe just a flow of bosons (instead of energy). At the moment, we neither intend or need to associate any specific energy to the current from some site to its adjacent independently of what normal mode is excited on the lattice.

### Thermal Equilibrium Currents

**Figure 2:**
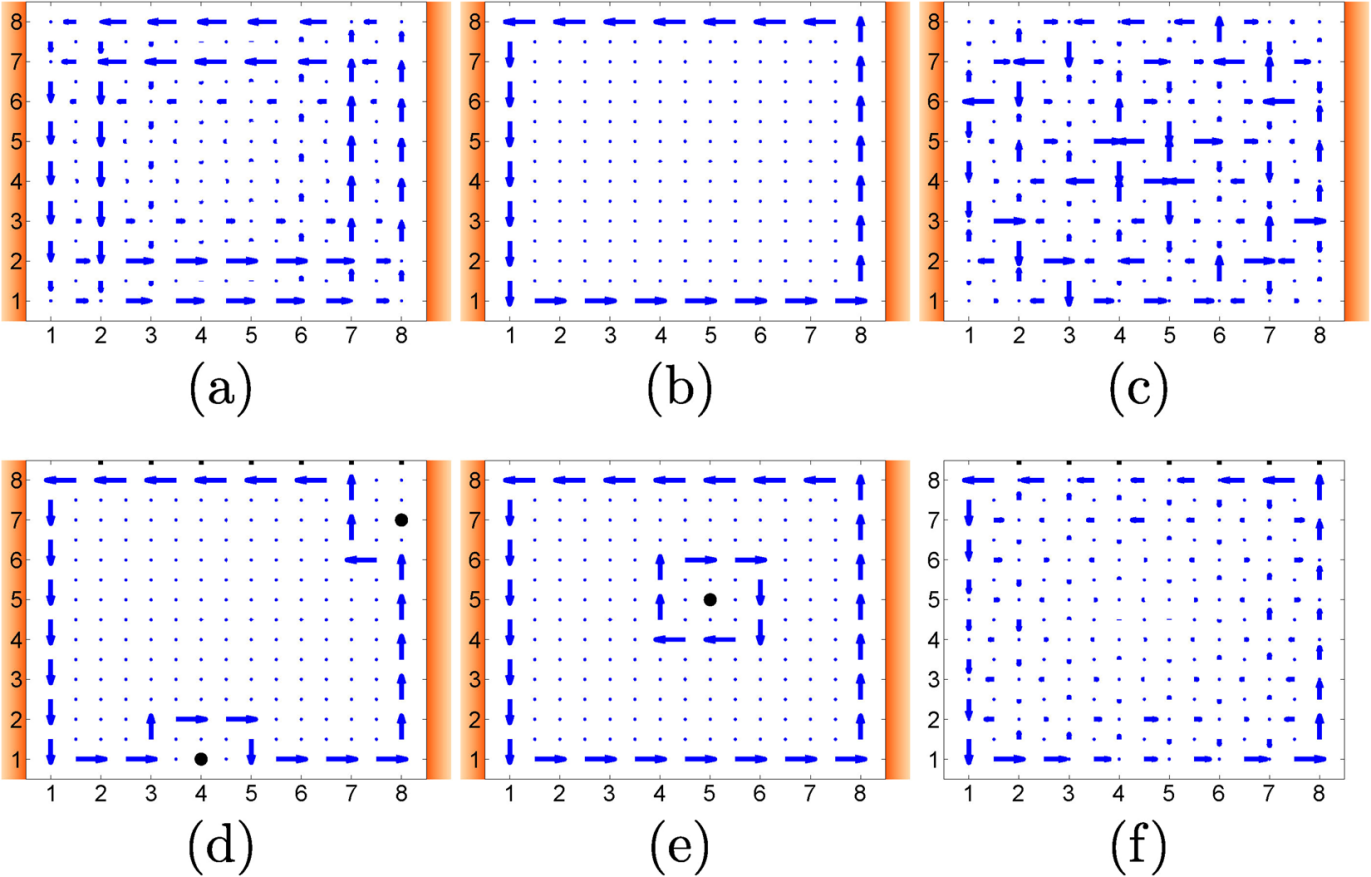
Current patterns for the system in contact with thermal baths at the same temperature *T*_*h*_ = *T*_*c*_ = *T*: (**a**) for *T* = 4 K, (**b**) for *T* = 2500 K, and (**c**) for *T* = 10^15^ K; and under the presence of different defects represented with a black dot in (**d**) and (**e**) for *T* = 2500 K. In (**f**) the pattern for an individual excited edge mode is depicted. For the sake of illustration the magnitude of the currents has been normalized in every subplot, see main text for specific numbers.

We shall move our attention to the behavior of the internal currents, refering to the external ones when appropriate. Motivated by realistic simulations^11^, we have taken *N* = 8 and *ω*_0_ = 193 THz, *J* = 2.6 THz and *α* = 0.15; different values may be considered leading to similar conclusions. In Fig. 2(a),(b) and (c) the current pattern on the lattice in the steady state limit is depicted as a function of bath temperature. For low temperatures ($\lesssim 70$ K for this choice of parameters) the ground state, which is a bulk state, is mainly populated and no edge current is observed. As temperature increases (in a range of 70–10^13^ K approximately), the edge states start populating and current start concentrating on the edge [Fig. 2(a) and (b)]. For high enough temperature everything gets mixed up [Fig. 2(c)].

Notably, the accumulation of current on the edge relative to the bulk is in fact higher for a thermal state at a suited temperature than for a single edge mode, Fig. 2(f). Specifically, for the aforementioned values in Fig. 2, the ratio between edge/bulk current is about 2 for 4 K, 10^3^ for 2500 K, 1 for 10^15^ K, and 10 for the individual edge mode (see Fig. S3 in the supplementary information document for a detailed plot). This TEE, the point (i) above, can be related to a combined action. On the one hand, the currents can be written as a sum $\langle {\mathcal{J}}\,\rangle ={\sum }_{k}{\bar{n}}_{k}(T){\langle {\mathcal{J}}\rangle }_{k}$ with ${\langle {\mathcal{J}}\rangle }_{k}$ the current for one excitation in the mode *k*. It is well known that the density of states of the energy spectrum is much higher for bulk states than for edge states (see Fig. S1 in the supplementary information document). Therefore the factor ${\bar{n}}_{k}(T)={\{\exp [\hslash {\omega }_{k}/({k}_{B}T)]-\mathrm{1\}}}^{-1}$ as a function *k* is flatter for values of *k* belonging to bulk modes (bulk currents) than for edge modes (edge currents), providing more mixing in the bulk than on the edge. On the other hand, the contribution of the edge states is amplified on the edges since they decay very rapidly into the bulk.

In addition, as anticipated in point (ii) above, we observe that the edge thermal current is robust under the presence of defects (TTC), Fig. 2(d) and (e). Defects are effectively created by far off-detuning of site local energies. Moreover, if a defect is allocated in the bulk, the system generates a current around it with opposite direction to the edge current Fig. 2(e). In this regard, it is worth to mention this is so despite the bulk has not direct contact with the baths, only left and right edges are in contact with them. Note that the presence of local currents in the equilibrium case does not lead to any thermodynamic inconsistency as the net heat flow as accounted for the external currents vanishes $\langle {{\mathcal{J}}}_{h}\rangle =\langle {{\mathcal{J}}}_{c}\rangle =0$.

### Nonthermal Equilibrium Currents

For the out-of-thermal-equilibrium situation, *T*_*h*_ > *T*_*c*_, an edge chiral current is also found. Actually, we obtain a similar pattern as for baths at the same temperature. However in this case, the current flows on one edge in the opposite direction to the heat. This apparent violation of the second law on one edge is a topological effect (ENC), and, as stated in point (iii) above, it is not a contradiction with thermodynamics as the total heat current as measured by external currents (${\langle {{\mathcal{J}}}_{h}\rangle }_{{\rm{ss}}}=-{\langle {{\mathcal{J}}}_{c}\rangle }_{{\rm{ss}}}=29.85$ MeV/s for this choice of parameters) does satisfy the second law. Yet, this feature of the edge current is very remarkable as it implies that a local measurement on an edge does not provide enough information to infer the positions of hot and cold baths in this topological system.

**Figure 3:**
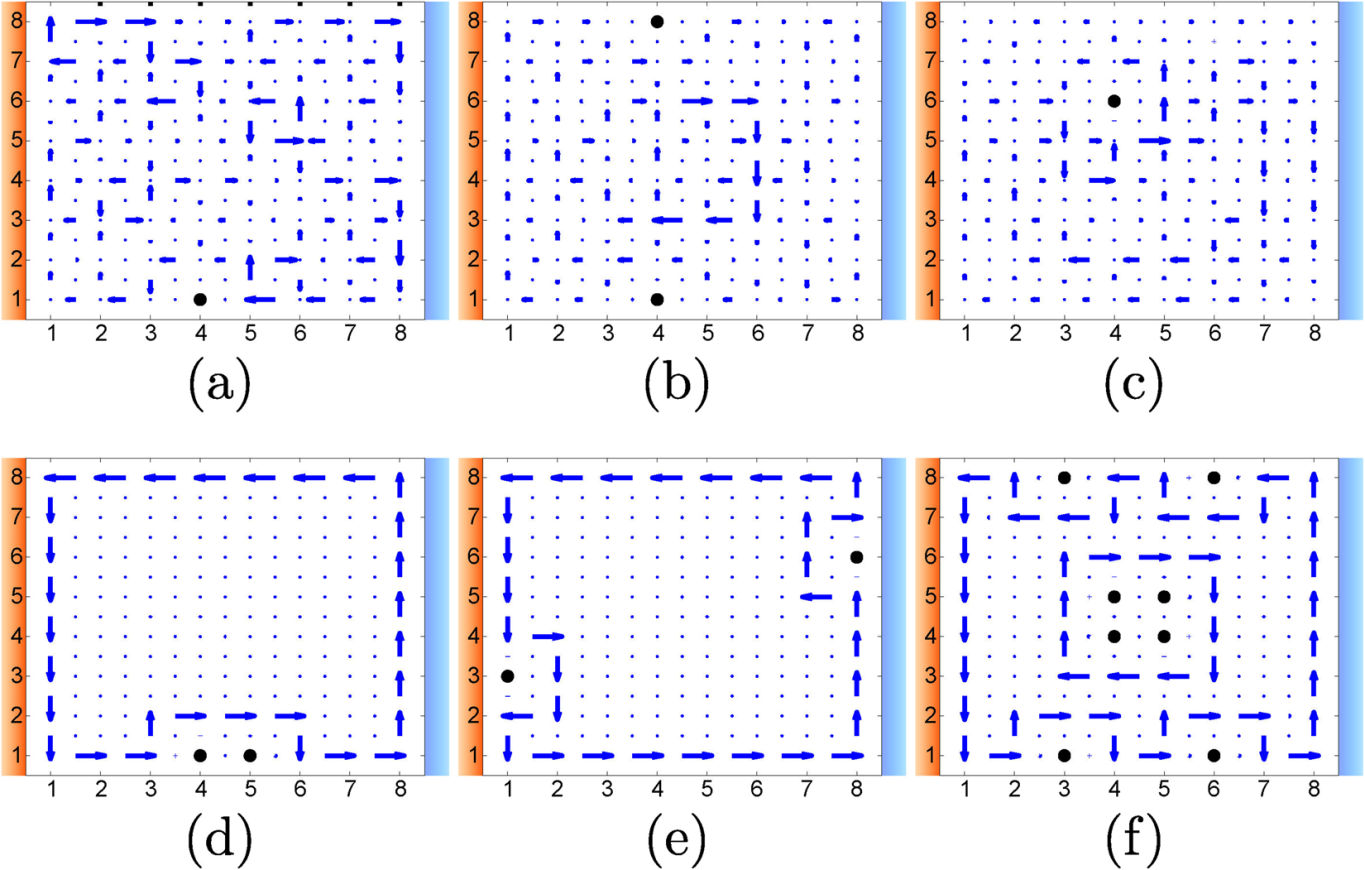
Current patterns for an out-of-equilibrium situation under the presence of defects indicated with a black dot (*T*_*h*_ = 2500 K and *T*_*c*_ = 1500 K, similar results are obtained for other values avoiding extremely high and low temperatures, see Fig. 2). In (**a**), (**b**) and (**c**) the distribution of defects respects neither ΘΣ_*y*_ nor *R*_*π*_ symmetries, and the currents become unstable. In contrast, in (**d**), (**e**) and (**f**) the distribution of defects is invariant under ΘΣ_*y*_, *R*_*π*_, and both ΘΣ_*y*_ and *R*_*π*_, respectively, and the currents remain robust.

The behavior of the nonthermal equilibrium current under the presence of impurities in the lattice exhibits novel and intriguing features. As aforementioned, just from the symmetries of the master equation (4), we may expect robustness for a distribution of defects satisfying either ΘΣ_*y*_ or *R*_*π*_. For a different situation, the result is far from clear. It turns out that the nonthermal equilibrium currents are indeed stable for defect distributions preserving these symmetries, see Fig. 3(d),(e) and (f). Nonetheless, currents become unstable for spatially distributed defects not complying with them, Fig. 3(a),(b) and (c).

**Figure 4:**
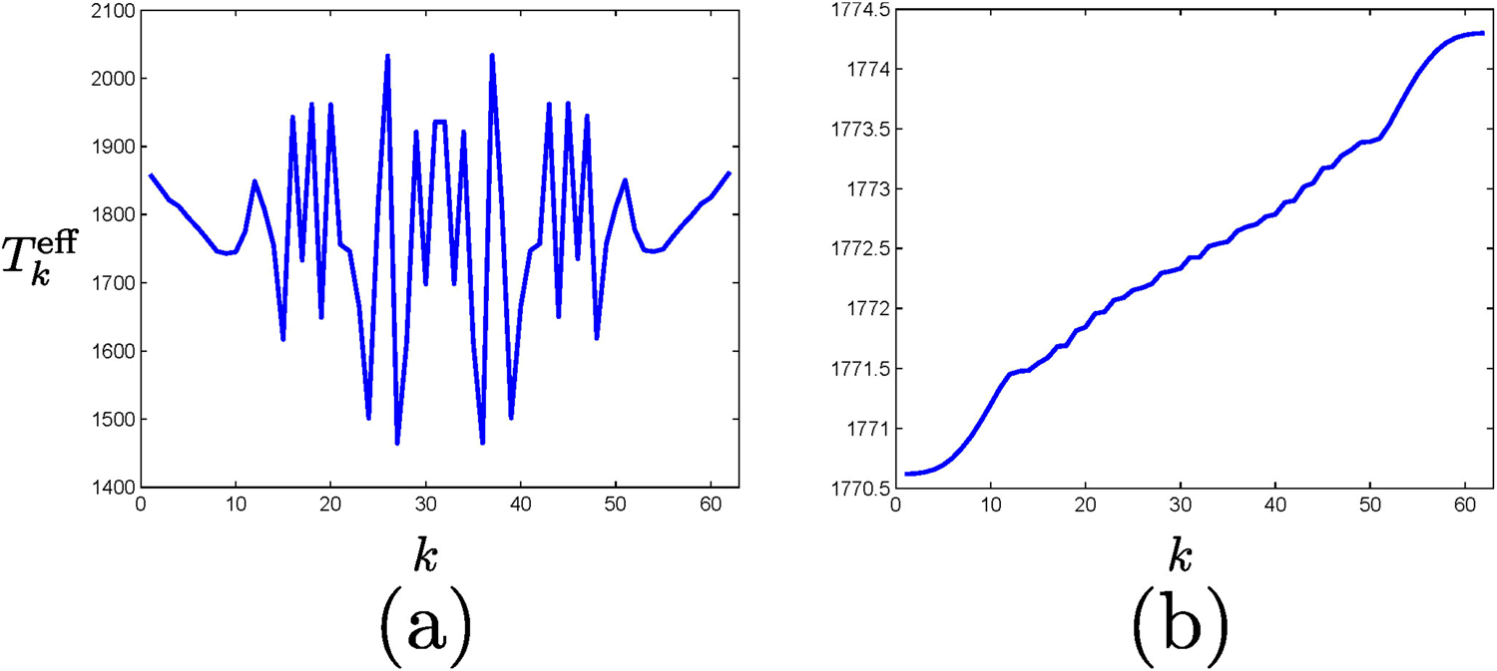
Dependence of ${T}_{k}^{{\rm{eff}}}$ on the specific mode *k* for: (**a**) an arbitrary distribution of defects, and (**b**) when either ΘΣ_*y*_ or *R*_*π*_ symmetry is satisfied. *T*_*h*_ = 2500 K and *T*_*c*_ = 1500 K.

The appearance of these surprising symmetry-protected currents in the nonthermal equilibrium situation can be explained in terms of the mode-dependent temperature ${T}_{k}^{{\rm{eff}}}$, Eq. (6). Because the oscillatory behavior of *s*_*k*_ and *r*_*k*_ [the amplitude of a mode on the left (*s*_*k*_) and right (*r*_*k*_) edges strongly varies with the mode], ${T}_{k}^{{\rm{eff}}}$ presents a fluctuating profile resulting in an effective increment of noise and mixedness that generally removes edge currents. The system does not resist such a high degree of noise. However, under invariance by either ΘΣ_*y*_ or *R*_*π*_, both coefficients are equal *s*_*k*_ = *r*_*k*_, and therefore ${T}_{k}^{{\rm{eff}}}$ becomes independent of *s*_*k*_ and *r*_*k*_, Eq. (6). This makes ${T}_{k}^{{\rm{eff}}}$ to be a monotonically increasing function of *ω*_*k*_, see Fig. 4, and there is a monotonic mode population with a similar pattern that in the thermal equilibrium situation: the more energy $\hslash {\omega }_{k}$, the less population in the mode, and edge currents are observed. Thus, the effect of disorder in the Liouvillian dynamics is minimal provided that the original symmetries are satisfied.

## Discussion

The interaction of a bosonic topological system with one and two thermal baths presents a rich and new phenomenology. For one single bath, we find a wide range of temperatures where topological edge heat currents are present despite thermal fluctuations. For two baths, the edge heat current presents further remarkable properties: it is still robust with respect to defect perturbations as long as these defects respect certain discrete global symmetries. Moreover, one topological current flows against the natural arrow of the heat according to the second law (without implying any violation).

The *selective* stability of the out-of-thermal-equilibrium current (SPNC) is due to a dissipative symmetry, or Liouvillian symmetry in the master equation (4), not to any system Hamiltonian symmetry (2). Specifically, the system presents a new kind of protection, the *dissipative symmetry*-*protection*, which minimizes the transition between the edge-conducting and the insulating phase in the steady state provided that some symmetries are satisfied. This is an open-system effect, similar to the usual Hamiltonian symmetry-protection of closed systems, where the transition probability between both states of matter is highly suppressed due to a symmetry property. This enforces the interest to study symmetry-protected topological ordered systems beyond the realm of Hamiltonian dynamics.

Moreover, note that in this case the property that an excitation or carrier has in order to overcome a defect is by no means understandable by some local argument. The excitation does or does not circulate around the defect depending on whether in other point of the lattice–which can be very far away–there is another defect such that a global symmetry of the lattice is preserved. This manifests unquestionably the topological physics implied in this current.

Our results are based on a master equation formalism valid under the standard condition of weak coupling between system and reservoirs, and it is particularly suited to describe the steady state regime. For the sake of comparison, another approach based on a local formalism for the master equations of many body systems is given in the supplementary information document, but it does not lead to results (i)–(iv).

Although the study is carried out for the emblematic Hofstadter model of bosons, similar conclusions are drawn for the same class of topological insulators. We have not focused our attention on some specific platform, but master equations usually adequate very good to quantum optical systems. Therefore, set-ups based on optical lattices^13–15^ or photonic systems^9–12, 24, 25^ seem the most indicated to experimentally observe the effects here reported.

Finally, the edge current instability for a particular class of defect distributions is not only a novel, fundamental and intriguing effect, but may also impose some restrictions when manufacturing quantum Hall systems if we expect them to be stable when subject to some temperature gradient. This is of particular importance for example in the topological transport of photons^9–12, 24, 25^, phonons^26–29^ or magnons^30–32^ as their neutral charge prevents them to be displaced by applying electric fields.

## Methods

### Master Equation

The bosonic Hofstadter Hamiltonian in general terms reads

13 $${H}_{S}=\sum _{x,y}\hslash {\omega }_{0}{a}_{x,y}^{\dagger }{a}_{x,y}+V,$$

with

14 $$V=-\hslash J\sum _{x,y}{a}_{x+\mathrm{1,}y}^{\dagger }{a}_{x,y}{e}^{i{\theta }_{x,y}^{X}}+{a}_{x,y+1}^{\dagger }{a}_{x,y}{e}^{i{\theta }_{x,y}^{Y}}+{\rm{h}}{\rm{.c}}{\rm{.}}$$

Here *a*_*x*, *y*_ stands for the bosonic operator on the site (*x*, *y*) of the *N* × *N* lattice and

15 $${\theta }_{x,y}^{X}={\int }_{x}^{x+1}{\boldsymbol{A}}\cdot dx,\quad {\rm{and}}\quad {\theta }_{x,y}^{Y}={\int }_{y}^{y+1}{\boldsymbol{A}}\cdot dy,$$

where ***A*** denotes a gauge field.

After diagonalization, Eq. (13) yields ${H}_{S}=\sum _{k}\hslash {\omega }_{k}{b}_{k}^{\dagger }{b}_{k}$, where *ω*_*k*_ is the frequency of the normal mode *k* and *a*_*x*, *y*_ = ∑_*k*_ *ψ*_*k*_(*x*, *y*)*b*_*k*_, with *ψ*_*k*_(*x*, *y*) the “one-particle” eigenfunctions. In terms of these eigenmodes the system-reservoir Hamiltonian can be written as

16 $${H}_{SR}=\sum _{j,k}\,{g}_{j}({L}_{j,k}{b}_{k}+{L}_{j,k}^{\dagger }{b}_{k}^{\dagger })+{g}_{j}({R}_{j,k}{b}_{k}+{R}_{j,k}^{\dagger }{b}_{k}^{\dagger })$$

with

17 $${L}_{j,k}\,:=\sum _{y}{\psi }_{k}\mathrm{(1,}\,y)({A}_{j,y}+{A}_{j,y}^{\dagger }),\quad {\rm{and}}\quad {R}_{j,k}\,:=\sum _{y}{\psi }_{k}(N,y)({B}_{j,y}+{B}_{j,y}^{\dagger }),$$

for operators of left and right reservoirs, respectively.

We obtain the Davies generator of the weak coupling limit^33, 34^ by applying the standard procedure (technical details are in the supplementary information document). The subsequent master equation reads

18 $$\begin{array}{c}\frac{d\rho }{dt}= {\mathcal L} (\rho )=-\frac{i}{\hslash }[{H}_{S},\rho ]+\sum _{k}{\gamma }_{k}\{{s}_{k}[{\bar{n}}_{k}({T}_{h})+\mathrm{1]}+{r}_{k}[{\bar{n}}_{k}({T}_{c})+\mathrm{1]\}}({b}_{k}\rho {b}_{k}^{\dagger }-\frac{1}{2}\{{b}_{k}^{\dagger }{b}_{k},\rho \})\\ \quad \quad \,+\,\sum _{k}{\gamma }_{k}[{s}_{k}{\bar{n}}_{k}({T}_{h})+{r}_{k}{\bar{n}}_{k}({T}_{c})]({b}_{k}^{\dagger }\rho {b}_{k}-\frac{1}{2}\{{b}_{k}{b}_{k}^{\dagger },\rho \}),\end{array}$$

where ${\bar{n}}_{k}(T)={\{\exp [\hslash {\omega }_{k}/({k}_{B}T)]-\mathrm{1\}}}^{-1}$ denotes the mean number of bosons with frequency *ω*_*k*_ and temperature *T*, and *γ*_*k*_ is a constant that depends on the strength of the coupling via the spectral density $f(\omega )\sim \sum \,{g}_{j}^{2}\delta ({\omega }_{j}-\omega )$. For the sake of simplicity we shall assume the same decay rate for each eigenmode *γ*_*k*_ = *γ*, although this is not relevant to our conclusions. Furthermore the remaining constants in the equation are given by ${s}_{k}={\sum }_{y=1}^{N}{|{\psi }_{k}\mathrm{(1,}y)|}^{2}$ and ${r}_{k}={\sum }_{y=1}^{N}{|{\psi }_{k}(N,y)|}^{2}$. In the thermal equilibrium situation *T*_*h*_ = *T*_*c*_ = *T*, it is well-known (see e.g.^35, 36^) this master equation describes the dynamics of the system towards thermal equilibrium with the baths, so that the steady state obtained for long times is ${\rho }_{\beta }=\frac{{e}^{-\beta {H}_{S}}}{Z}$ with *β* = 1/(*k*_*B*_ *T*) and *Z* = Tr[exp(−*βH*_*S*_)].

For the general nonequilibrium case *T*_*h*_ > *T*_*c*_, we note that the master equation (18) can be rewritten as

19 $$\begin{array}{c}\frac{d\rho }{dt}= {\mathcal L} (\rho )=-\frac{i}{\hslash }[{H}_{S},\rho ]+\sum _{k}\gamma [{\bar{n}}_{k}({T}_{k}^{{\rm{eff}}})+1]({b}_{k}\rho {b}_{k}^{\dagger }-\frac{1}{2}\{{b}_{k}^{\dagger }{b}_{k},\rho \})\\ \quad \quad \quad \quad \quad \quad +\,\sum _{k}\gamma {\bar{n}}_{k}({T}_{k}^{{\rm{eff}}})({b}_{k}^{\dagger }\rho {b}_{k}-\frac{1}{2}\{{b}_{k}{b}_{k}^{\dagger },\rho \}),\end{array}$$

with an “effective” temperature ${T}_{k}^{{\rm{eff}}}$ depending on the mode and given by Eq. (6). Therefore, we conclude that the steady state is of the form:

20 $${\rho }_{{\rm{s}}{\rm{s}}}\,:=\mathop{{\rm{l}}{\rm{i}}{\rm{m}}}\limits_{t\to {\rm{\infty }}}\rho (t)={W}^{-1}\exp (-\sum _{k}\frac{\hslash {\omega }_{k}}{{k}_{B}{T}_{k}^{{\rm{e}}{\rm{f}}{\rm{f}}}}{b}_{k}^{\dagger }{b}_{k}),$$

with $$W={\rm{T}}{\rm{r}}[\exp (-{\sum }_{k}\frac{\hslash {\omega }_{k}}{{k}_{B}{T}_{k}^{{\rm{e}}{\rm{f}}{\rm{f}}}}{b}_{k}^{\dagger }{b}_{k})]$$.

### Current Operators

External and internal currents are derived from continuity equations. For the external case, using the master equation (18) we have

21 $$\frac{d\langle {H}_{S}\rangle }{dt}={\rm{T}}{\rm{r}}({H}_{S}\frac{d\rho }{dt})={\rm{T}}{\rm{r}}[{H}_{S}{\mathcal{L}}(\rho )]=\langle {{\mathcal{L}}}^{{\rm{\#}}}({H}_{S})\rangle =\langle {{\mathcal{J}}}_{h}\rangle +\langle {{\mathcal{J}}}_{c}\rangle ,$$

where ${{\mathcal{J}}}_{h}({{\mathcal{J}}}_{c})$ is the current operator that describes the heat flux between system and hot (cold) bath, and ${ {\mathcal L} }^{\#}$ denotes the Liouvillian at Eq. (18) in the Heisenberg picture. On the other hand, since

22 $${ {\mathcal L} }^{\#}({H}_{S})={ {\mathcal L} }_{c}^{\#}({H}_{S})+{ {\mathcal L} }_{h}^{\#}({H}_{S}),$$

with

23 $$\begin{array}{lll}{ {\mathcal L} }_{h}^{\#}({H}_{S}) & := & \sum _{k}{\gamma }_{k}\{{s}_{k}[{\bar{n}}_{k}({T}_{h})+\mathrm{1]\}}({b}_{k}^{\dagger }{H}_{S}{b}_{k}-\frac{1}{2}\{{b}_{k}^{\dagger }{b}_{k},{H}_{S}\})\\  &  & +\sum _{k}{\gamma }_{k}[{s}_{k}{\bar{n}}_{k}({T}_{h})]({b}_{k}{H}_{S}{b}_{k}^{\dagger }-\frac{1}{2}\{{b}_{k}{b}_{k}^{\dagger },{H}_{S}\})\\  & = & -\hslash \sum _{k}{\omega }_{k}{\gamma }_{k}{s}_{k}[{b}_{k}^{\dagger }{b}_{k}-{\bar{n}}_{k}({T}_{h})],\end{array}$$

and

24 $$\begin{array}{lll}{ {\mathcal L} }_{c}^{\#}({H}_{S}) & := & \sum _{k}{\gamma }_{k}\{{r}_{k}[{\bar{n}}_{k}({T}_{c})+\mathrm{1]\}}({b}_{k}^{\dagger }{H}_{S}{b}_{k}-\frac{1}{2}\{{b}_{k}^{\dagger }{b}_{k},{H}_{S}\})\\  &  & +\sum _{k}{\gamma }_{k}[{r}_{k}{\bar{n}}_{k}({T}_{c})]({b}_{k}{H}_{S}{b}_{k}^{\dagger }-\frac{1}{2}\{{b}_{k}{b}_{k}^{\dagger },{H}_{S}\})\\  & = & -\,\hslash \sum _{k}{\omega }_{k}{\gamma }_{k}{r}_{k}[{b}_{k}^{\dagger }{b}_{k}-{\bar{n}}_{k}({T}_{c})],\end{array}$$

we identify the external currents as

25 $${{\mathcal{J}}}_{h}\,:=-\hslash \sum _{k}{\omega }_{k}{\gamma }_{k}{s}_{k}[{b}_{k}^{\dagger }{b}_{k}-{\bar{n}}_{k}({T}_{h})],$$

26 $${{\mathcal{J}}}_{c}\,:=-\hslash \sum _{k}{\omega }_{k}{\gamma }_{k}{r}_{k}[{b}_{k}^{\dagger }{b}_{k}-{\bar{n}}_{k}({T}_{c})]\mathrm{.}$$

This identification is standard in the theory of open quantum systems, and it can be proven^37^ that the time-evolution described by the master equation (18) fulfills the entropy production inequality:

27 $$\frac{d{\mathcal{S}}}{dt}-\frac{\langle {{\mathcal{J}}}_{h}\rangle }{{T}_{h}}-\frac{\langle {{\mathcal{J}}}_{c}\rangle }{{T}_{c}}\ge \mathrm{0,}$$

where ${\mathcal{S}}=-{k}_{B}{\rm{Tr}}(\rho \,\mathrm{log}\,\rho )$ is the thermodynamical entropy.

In order to derive internal currents we make use of the exact form of the continuity equation and the Davies’ theorem^33^. Specifically, the exact equation for the population at the site (*x*, *y*) is given by

28 $$\begin{array}{rcl}\frac{d\langle {a}_{x,y}^{\dagger }{a}_{x,y}\rangle }{dt} & = & \frac{i}{\hslash }\langle [{H}_{S},{a}_{x,y}^{\dagger }{a}_{x,y}]\rangle +\frac{i}{\hslash }\langle [{H}_{SB},{a}_{x,y}^{\dagger }{a}_{x,y}]\rangle \\  & = & -iJ\langle {a}_{x+\mathrm{1,}y}^{\dagger }{a}_{x,y}{e}^{i{\theta }_{x,y}^{X}}-{a}_{x,y}^{\dagger }{a}_{x+\mathrm{1,}y}{e}^{-i{\theta }_{x,y}^{X}}\rangle \\  &  & -iJ\langle {a}_{x-\mathrm{1,}y}^{\dagger }{a}_{x,y}{e}^{-i{\theta }_{x,y}^{X}}-{a}_{x,y}^{\dagger }{a}_{x-\mathrm{1,}y}{e}^{i{\theta }_{x,y}^{X}}\rangle \\  &  & -iJ\langle {a}_{x,y+1}^{\dagger }{a}_{x,y}{e}^{i{\theta }_{x,y}^{Y}}-{a}_{x,y}^{\dagger }{a}_{x,y+1}{e}^{-i{\theta }_{x,y}^{Y}}\rangle \\  &  & -iJ\langle {a}_{x,y-1}^{\dagger }{a}_{x,y}{e}^{-i{\theta }_{x,y}^{Y}}-{a}_{x,y}^{\dagger }{a}_{x,y-1}{e}^{i{\theta }_{x,y}^{Y}}\rangle \\  &  & +\,\frac{i}{\hslash }\langle [{H}_{SB},{a}_{x,y}^{\dagger }{a}_{x,y}]\rangle \mathrm{.}\end{array}$$

Then, the (internal) current operators are identified as:

29 $${{\mathcal{J}}}_{(\to x),y}=iJ({a}_{x,y}^{\dagger }{a}_{x-\mathrm{1,}y}{e}^{i{\theta }_{x,y}^{X}}-{a}_{x-\mathrm{1,}y}^{\dagger }{a}_{x,y}{e}^{-i{\theta }_{x,y}^{X}}),$$

30 $${{\mathcal{J}}}_{x,(\to y)}=iJ({a}_{x,y}^{\dagger }{a}_{x,y-1}{e}^{i{\theta }_{x,y}^{Y}}-{a}_{x,y-1}^{\dagger }{a}_{x,y}{e}^{-i{\theta }_{x,y}^{Y}}),$$

where the subindex (→*x*) is a short notation for (*x* − 1 → *x*). So that ${{\mathcal{J}}}_{(\to x),y}$ denotes the operator for the current leaving the site (*x* − 1, *y*) and entering in (*x*, *y*). Similarly for (→*y*).

The term $\frac{i}{\hslash }\langle [{H}_{SB},{a}_{x,y}^{\dagger }{a}_{x,y}]\rangle$ in (28) is not zero only for *x* = 1 and *x* = *N*, and defines the exact external currents. Of course we cannot compute the exact time derivative $d\langle {a}_{x,y}^{\dagger }{a}_{x,y}\rangle /dt$; our approximation to it is given by the master equation (18). In fact, the Davies theorem^33^ asserts that the dissipative part of (18) is actually a weak coupling approximation of the term Tr_*B*_(−*i*[*H*_*SB*_, *ρ*]) (see the supplementary information document for further details). This means that, in a weak coupling regime, it is consistent to take the above exact internal currents operators as internal current operators also in the master equation approximation.

In a Landau-type gauge taken throughout the main document, we write ***A*** = (−|***B***|*y*, 0, 0) and internal currents take the form of Eqs (11) and (12), where *α* denotes the flux of the ***B*** field per unit cell.

More details about the derivation of the master equation and currents operators are provided in the supplementary information document.

## Electronic supplementary material

Supplementary Information

## References

[1] Hasan MZ, Kane CL (2010). Colloquium: Topological insulators. Rev. Mod. Phys..

[2] Qi XL, Zhang SC (2011). Topological insulators and superconductors. Rev. Mod. Phys..

[3] Moore JE (2010). The birth of topological insulators. Nature.

[4] Bernevig, B. A. and Hughes, T. L. *Topological Insulators and Topological Superconductors* (New Jersey: Princeton University Press, 2013).

[5] Jaksch D, Zoller P (2003). Creation of effective magnetic fields in optical lattices: the Hofstadter butterfly for cold neutral atoms. New J. Phys..

[6] Osterloh K, Baig M, Santos L, Zoller P, Lewenstein M (2005). Cold Atoms in Non-Abelian Gauge Potentials: From the Hofstadter “Moth” to Lattice Gauge Theory. Phys. Rev. Lett..

[7] Mazza L (2012). An optical-lattice-based quantum simulator for relativistic field theories and topological insulators. New J. Phys..

[8] Haldane FDM, Raghu S (2008). Possible Realization of Directional Optical Waveguides in Photonic Crystals with Broken Time-Reversal Symmetry. Phys. Rev. Lett..

[9] Hafezi M, Demler EA, Lukin MD, Taylor JM (2011). Robust optical delay lines with topological protection. Nature Phys..

[10] Rechtsman MC (2013). Photonic Floquet topological insulators. Nature.

[11] Hafezi M, Mittal S, Fan J, Migdall A, Taylor JM (2013). Imaging topological edge states in silicon photonics. Nature Photon..

[12] Khanikaev AB (2013). Photonic topological insulators. Nature Mater..

[13] Aidelsburger M (2013). Realization of the Hofstadter Hamiltonian with Ultracold Atoms in Optical Lattices. Phys. Rev. Lett..

[14] Miyake H, Siviloglou GA, Kennedy CJ, Burton WC, Ketterle W (2013). Realizing the Harper Hamiltonian with Laser-Assisted Tunneling in Optical Lattices. Phys. Rev. Lett..

[15] Stuhl BK, Lu H-I, Aycock LM, Genkina D, Spielman IB (2015). Visualizing edge states with an atomic Bose gas in the quantum Hall regime. Science.

[16] Viyuela O, Rivas A, Martin-Delgado MA (2012). Thermal instability of protected end states in a one-dimensional topological insulator. Phys. Rev. B.

[17] Rivas A, Viyuela O, Martin-Delgado MA (2013). Density-matrix Chern insulators: Finite-temperature generalization of topological insulators. Phys. Rev. B.

[18] Mazza L, Rizzi M, Lukin MD, Cirac JI (2013). Robustness of quantum memories based on Majorana zero modes. Phys. Rev. B.

[19] Hu Y, Baranov MA (2015). Effects of gapless bosonic fluctuations on Majorana fermions in an atomic wire coupled to a molecular reservoir. Phys. Rev. A.

[20] Linzner D, Wawer L, Grusdt F, Fleischhauer M (2016). Reservoir-induced Thouless pumping and symmetry-protected topological order in open quantum chains. Phys. Rev. B.

[21] Albert VV, Bradlyn B, Fraas M, Jiang L (2016). Geometry and Response of Lindbladians. Phys. Rev. X.

[22] Lieb EH, Yngvason J (1999). The physics and mathematics of the second law of thermodynamics. Phys. Rep..

[23] Hofstadter DR (1976). Energy levels and wave functions of Bloch electrons in rational and irrational magnetic fields. Phys. Rev. B.

[24] Wang Z, Chong Y, Joannopoulos JD, Soljačić M (2009). Observation of unidirectional backscattering-immune topological electromagnetic states. Nature.

[25] Lu L, Joannopoulos JD, Soljačić M (2014). Topological photonics. Nature Photon..

[26] Strohm C, Rikken GLJA, Wyder P (2005). Phenomenological Evidence for the Phonon Hall Effect. Phys. Rev. Lett..

[27] Zhang L, Ren J, Wang J-S, Li B (2010). Topological Nature of the Phonon Hall Effect. Phys. Rev. Lett..

[28] Li N (2012). Colloquium: Phononics: Manipulating heat flow with electronic analogs and beyond. Rev. Mod. Phys..

[29] Bermudez A, Schaetz T, Porras D (2011). Synthetic Gauge Fields for Vibrational Excitations of Trapped Ions. Phys. Rev. Lett..

[30] Katsura H, Nagaosa N, Lee PA (2010). Theory of the Thermal Hall Effect in Quantum Magnets. Phys. Rev. Lett..

[31] Onose SY (2010). Observation of the Magnon Hall Effect. Science.

[32] Zhang L, Ren J, Wang J-S, Li B (2013). Topological magnon insulator in insulating ferromagnet. Phys. Rev. B.

[33] Davies EB (1974). Markovian master equations. Commun. Math. Phys..

[34] Davies EB (1976). Markovian master equations. II. Math. Ann..

[35] Alicki, R. and Lendi, K. *Quantum Dynamical Semigroups and Applications* (Berlin: Springer, 2007).

[36] Rivas, A. and Huelga, S. F. *Open Quantum Systems*. *An Introduction* (Heidelberg: Springer, 2012).

[37] Spohn H (1978). Entropy production for quantum dynamical semigroups. J. Math. Phys..

