# Supplementary material for: Topological Heat Transport and Symmetry-Protected Boson Currents
Source: Sci Rep. 2017 Jul 25;7:6350. doi: 10.1038/s41598-017-06722-x (PMC5526937; doi:10.1038/s41598-017-06722-x)
Supplement: Supplementary file 1 — Supplementary Information [file 41598_2017_6722_MOESM1_ESM.pdf]

Supplementary Information: Topological Heat Transport and Symmetry-Protected Boson Currents

Ángel Rivas and Miguel A. Martin-Delgado
Departamento de Física Teórica I, Universidad Complutense, 28040 Madrid, Spain.
CCS -Center for Computational Simulation, Campus de Montegancedo UPM, 28660 Boadilla del Monte, Madrid, Spain.

MASTER EQUATION

The bosonic Hofstadter Hamiltonian in general terms reads

$$H_S = \sum_{x,y} \hbar\omega_0 a^\dagger_{x,y} a_{x,y} + V, \tag{S1}$$

with

$$V = -\hbar J \sum_{x,y} a^\dagger_{x+1,y} a_{x,y} e^{i\theta^X_{x,y}} + a^\dagger_{x,y+1} a_{x,y} e^{i\theta^Y_{x,y}} + \text{h.c.} \tag{S2}$$

Here $a_{x,y}$ stands for the bosonic operator on the site (x,y) of the $N \times N$ lattice and

$$\theta^X_{x,y} = \int_x^{x+1} \boldsymbol{A}\cdot d\boldsymbol{x}, \quad \text{and} \quad \theta^Y_{x,y} = \int_y^{y+1} \boldsymbol{A}\cdot d\boldsymbol{y}, \tag{S3}$$

where $\boldsymbol{A}$ denotes a gauge field.

The Hamiltonian Eq. (S1) can be written in matrix form as

$$H_S = \Psi^\dagger \mathbf{H_S} \Psi \tag{S4}$$

where $\Psi = (a_{1,1}, \dots, a_{N,1}, a_{1,2}, \dots, a_{N,2}, \dots, a_{1,N}, \dots, a_{N,N})^{\mathrm{t}}$ and

$$\mathbf{H_S} = \hbar \begin{pmatrix} M_1 & I_1^\dagger & & \\ I_1 & M_2 & \ddots & \\ & \ddots & \ddots & I^\dagger_{N-1} \\ & & I_{N-1} & M_N \end{pmatrix}, \tag{S5}$$

with $N \times N$ matrices

$$I_n = -J \begin{pmatrix} e^{i\theta^Y_{1,n}} & & \\ & \ddots & \\ & & e^{i\theta^Y_{N,n}} \end{pmatrix} \quad \text{and} \quad M_n = \begin{pmatrix} \omega_0 & -Je^{-i\theta^X_{1,n}} & & \\ -Je^{i\theta^X_{1,n}} & \ddots & \ddots & \\ & \ddots & \ddots & -Je^{-i\theta^X_{N-1,n}} \\ & & -Je^{i\theta^X_{N-1,n}} & \omega_0 \end{pmatrix}, \tag{S6}$$

so that $\mathbf{H_S}$ is a $N^2 \times N^2$ matrix.

By diagonalizing the matrix $\mathbf{H_S} = \mathbf{U}\mathbf{D}\mathbf{U}^\dagger$ we obtain the spectrum of H_S which can be visualized as the celebrated Hofstadter butterfly [S1]. The eigenmodes of this Hamiltonian are given by $\boldsymbol{b} = \mathbf{U}^\dagger\Psi$, with $\boldsymbol{b} = (b_1, b_2, \dots, b_{N^2})^{\mathrm{t}}$. In Fig. S1 we have represented the density of states for an 8×8 lattice, the low density regions correspond to energies associated with edge eigenmodes.

In terms of these eigenmodes the system-reservoir Hamiltonian yields

$$\begin{aligned} H_{SR} &= \sum_{j,y} g_j (A_{j,y} + A^\dagger_{j,y})(a_{1,y} + a^\dagger_{1,y}) + g_j (B_{j,y} + B^\dagger_{j,y})(a_{N,y} + a^\dagger_{N,y}) \\ &= \sum_{j,y} g_j (A_{j,y} + A^\dagger_{j,y}) \left(\sum_k u_{N(y-1)+1,k} b_k + u^*_{N(y-1)+1,k} b^\dagger_k \right) + g_j (B_{j,y} + B^\dagger_{j,y}) \left(\sum_k u_{Ny,k} b_k + u^*_{Ny,k} b^\dagger_k \right), \end{aligned} \tag{S7}$$

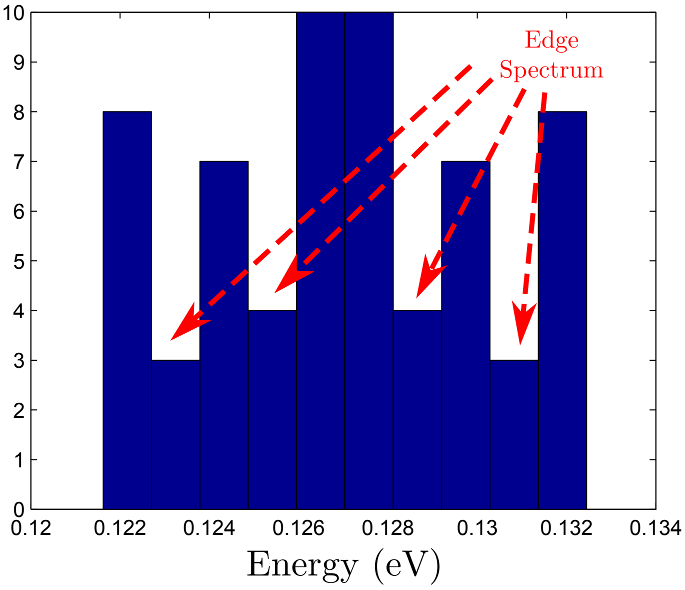

FIG. S1: Density of states for an 8×8 lattice ($\omega_0 = 193$ THz and $J = 2.6$ THz). The four low density regions correspond to energies of edge eigenmodes.

where we have taken into account that the mode $a_{x,y}$ is allocated as the component $N(y-1)+x$ of the vector Ψ and $u_{i,j}$ denotes the components of the unitary matrix $\mathbf{U}$. After some rearrangement, we write

$$H_{SR} = \sum_{j,k} g_j \left(L_{j,k} b_k + L^\dagger_{j,k} b^\dagger_k \right) + g_j \left(R_{j,k} b_k + R^\dagger_{j,k} b^\dagger_k \right) \tag{S8}$$

with

$$L_{j,k} := \sum_y u_{N(y-1)+1,k} (A_{j,y} + A^\dagger_{j,y}), \quad \text{and} \quad R_{j,k} := \sum_y u_{Ny,k} (B_{j,y} + B^\dagger_{j,y}), \tag{S9}$$

for operators of left and right reservoirs, respectively.

Now, we obtain the Davies generator of the weak coupling limit [S2] by applying the standard procedure (see, for instance, [S3]). Note that there are not mixed terms between different site reservoirs in the correlation functions, so the second order dissipator becomes

$$\mathcal{D}(\rho) = \sum_k \int_{-\infty}^{\infty} d\tau \sum_j g_j^2 e^{i\omega_k \tau} \left(\langle \tilde{L}^\dagger_{j,k}(\tau) L_{j,k} \rangle_{\beta_h} + \langle \tilde{R}^\dagger_{j,k}(\tau) R_{j,k} \rangle_{\beta_c} \right) [b_k \rho b^\dagger_k - \frac{1}{2}\{b^\dagger_k b_k, \rho\}]$$
$$+ g_j^2 e^{-i\omega_k \tau} \left(\langle \tilde{L}_{j,k}(\tau) L^\dagger_{j,k} \rangle_{\beta_h} + \langle \tilde{R}_{j,k}(\tau) R^\dagger_{j,k} \rangle_{\beta_c} \right) [b^\dagger_k \rho b_k - \frac{1}{2}\{b_k b^\dagger_k, \rho\}], \tag{S10}$$

where $\tilde{L}_{j,k}(\tau) = \sum_y u_{N(y-1)+1,k} [A_{j,y} \exp(-i\nu_{j,y}\tau) + A^\dagger_{j,y} \exp(i\nu_{j,y}\tau)]$, and (similarly for) $\tilde{R}_{j,k}(\tau)$, are the operators $L_{j,k}$ and $R_{j,k}$ in the interaction picture ($\nu_{j,y}$ is the frequency of the bath mode $A_{j,y}$), ω_k is the frequency associated to the eigenmode b_k, and $\beta_{h,c} = 1/(k_B T_{h,c})$ are inverse temperatures of hot and cold baths. Following the usual steps for the derivation [S3], we obtain the following master equation:

$$\frac{d\rho}{dt} = \mathcal{L}(\rho) = -\frac{i}{\hbar}[H_S, \rho] + \sum_k \gamma_k \left\{ s_k [\bar{n}_k(T_h) + 1] + r_k [\bar{n}_k(T_c) + 1] \right\} \left(b_k \rho b^\dagger_k - \frac{1}{2}\{b^\dagger_k b_k, \rho\} \right)$$
$$+ \sum_k \gamma_k \left[s_k \bar{n}_k(T_h) + r_k \bar{n}_k(T_c) \right] \left(b^\dagger_k \rho b_k - \frac{1}{2}\{b_k b^\dagger_k, \rho\} \right), \tag{S11}$$

where $\bar{n}_k(T) = \{\exp[\hbar\omega_k/(k_B T)] - 1\}^{-1}$ denotes the mean number of bosons with frequency ω_k and temperature T, and γ_k is a constant that depends on the strength of the coupling via the spectral density $f(\omega) \sim \sum g_j^2 \delta(\omega_j - \omega)$. For

the sake of simplicity we have assumed in the main text the same decay rate for each eigenmode $\gamma_k = \gamma$, although this is not relevant to our conclusions. Furthermore the constants s_k and r_k are related to the matrix $\mathbf{U}$ via:

$$s_k = \sum_{y=1}^{N} [\mathbf{U}^\dagger]_{k,N(y-1)+1} [\mathbf{U}]_{N(y-1)+1,k} = \sum_{y=1}^{N} |u_{N(y-1)+1,k}|^2, \tag{S12}$$

$$r_k = \sum_{y=1}^{N} [\mathbf{U}^\dagger]_{k,Ny} [\mathbf{U}]_{Ny,k} = \sum_{x=1}^{N} |u_{Ny,k}|^2. \tag{S13}$$

Since the columns of the matrix $\mathbf{U}$ are the coordinates in real space of "one-particle" wavefunctions $\psi_k(x, y)$, after reordering, we obtain

$$s_k = \sum_{y=1}^{N} |\psi_k(1, y)|^2, \quad \text{and} \quad r_k = \sum_{y=1}^{N} |\psi_k(N, y)|^2. \tag{S14}$$

In the thermal equilibrium situation $T_h = T_c = T$, the master equation (S11) becomes

$$\frac{d\rho}{dt} = \mathcal{L}(\rho) = -\frac{i}{\hbar}[H_S, \rho] + \sum_k \bar{\gamma}_k [\bar{n}_k(T) + 1] \Big(b_k \rho b_k^\dagger - \frac{1}{2}\{b_k^\dagger b_k, \rho\} \Big) + \sum_k \bar{\gamma}_k \bar{n}_k(T) \Big(b_k^\dagger \rho b_k - \frac{1}{2}\{b_k b_k^\dagger, \rho\} \Big), \tag{S15}$$

with $\bar{\gamma}_k = \gamma(s_k + r_k)$. This equation describes the dynamics of the system towards thermal equilibrium with the baths, so that the steady state obtained for long times is the Gibbs state at the same temperature as the baths:

$$\rho_{\text{ss}} := \lim_{t\to\infty} \rho(t) = \rho_\beta = \frac{e^{-\beta H_S}}{Z}, \tag{S16}$$

with $\beta = 1/(k_B T)$ and $Z = \text{Tr}[\exp(-\beta H_S)]$.

In the general nonequilibrium case $T_h > T_c$, it is usually involved to obtain the steady state at the stationary limit. However, in this case we can find it by noting that the master equation (S11) can be rewritten as

$$\frac{d\rho}{dt} = \mathcal{L}(\rho) = -\frac{i}{\hbar}[H_S, \rho] + \sum_k \gamma [\bar{n}_k(T_k^{\text{eff}}) + 1] \Big(b_k \rho b_k^\dagger - \frac{1}{2}\{b_k^\dagger b_k, \rho\} \Big) + \sum_k \gamma \bar{n}_k(T_k^{\text{eff}}) \Big(b_k^\dagger \rho b_k - \frac{1}{2}\{b_k b_k^\dagger, \rho\} \Big), \tag{S17}$$

with some "effective" temperature T_k^{eff} depending on the mode:

$$T_k^{\text{eff}} := \frac{\hbar\omega_k}{k_B \log\left\{ \frac{\exp\left(\frac{\hbar\omega_k}{k_B T_h}\right)\left[\exp\left(\frac{\hbar\omega_k}{k_B T_c}\right)-1\right] s_k + \exp\left(\frac{\hbar\omega_k}{k_B T_c}\right)\left[\exp\left(\frac{\hbar\omega_k}{k_B T_h}\right)-1\right] r_k}{\left[\exp\left(\frac{\hbar\omega_k}{k_B T_c}\right)-1\right] s_k + \left[\exp\left(\frac{\hbar\omega_k}{k_B T_h}\right)-1\right] r_k} \right\}}. \tag{S18}$$

Therefore, the master equation (S17) is the same as the one describing the dynamics of a collection of N^2 independent modes with different frequencies interacting with N^2 thermal baths with different temperatures T_k^{eff}. Hence, we conclude that the steady state is of the form:

$$\rho_{\text{ss}} := \lim_{t\to\infty} \rho(t) = W^{-1} \exp\left(-\sum_k \frac{\hbar\omega_k}{k_B T_k^{\text{eff}}} b_k^\dagger b_k \right), \tag{S19}$$

with $W = \text{Tr}\left[\exp\left(-\sum_k \frac{\hbar\omega_k}{k_B T_k^{\text{eff}}} b_k^\dagger b_k\right)\right]$.

CURRENT OPERATORS

External and internal currents are derived from continuity equations. For the external case, using the master equation (S11) we have

$$\frac{d\langle H_S\rangle}{dt} = \mathrm{Tr}\left(H_S\frac{d\rho}{dt}\right) = \mathrm{Tr}\left[H_S\mathcal{L}(\rho)\right] = \langle\mathcal{L}^\sharp(H_S)\rangle = \langle\mathcal{J}_h\rangle + \langle\mathcal{J}_c\rangle, \tag{S20}$$

where $\mathcal{J}_h$ ($\mathcal{J}_c$) is the current operator that describes the heat flux between system and hot (cold) bath, and $\mathcal{L}^\sharp$ denotes the Liouvillian at Eq. (S11) in the Heisenberg picture. Note that in the usual convention a positive current (meaning an increment of $\langle H_S\rangle$) is associated to energy flowing from outside to the system, whereas a negative current describes energy flowing from system to outside. On the other hand, since

$$\mathcal{L}^\sharp(H_S) = \mathcal{L}^\sharp_c(H_S) + \mathcal{L}^\sharp_h(H_S), \tag{S21}$$

with

$$\begin{aligned}\mathcal{L}^\sharp_h(H_S) := &\sum_k \gamma_k\{s_k[\bar{n}_k(T_h)+1]\}\left(b_k^\dagger H_S b_k - \frac{1}{2}\{b_k^\dagger b_k, H_S\}\right)\\ &+\sum_k \gamma_k\left[s_k\bar{n}_k(T_h)\right]\left(b_k H_S b_k^\dagger - \frac{1}{2}\{b_k b_k^\dagger, H_S\}\right) = -\hbar\sum_k \omega_k\gamma_k s_k[b_k^\dagger b_k - \bar{n}_k(T_h)],\end{aligned} \tag{S22}$$

and

$$\begin{aligned}\mathcal{L}^\sharp_c(H_S) := &\sum_k \gamma_k\{r_k[\bar{n}_k(T_c)+1]\}\left(b_k^\dagger H_S b_k - \frac{1}{2}\{b_k^\dagger b_k, H_S\}\right)\\ &+\sum_k \gamma_k\left[r_k\bar{n}_k(T_c)\right]\left(b_k H_S b_k^\dagger - \frac{1}{2}\{b_k b_k^\dagger, H_S\}\right) = -\hbar\sum_k \omega_k\gamma_k r_k[b_k^\dagger b_k - \bar{n}_k(T_c)],\end{aligned} \tag{S23}$$

we identify the currents as

$$\mathcal{J}_h := -\hbar\sum_k \omega_k\gamma_k s_k[b_k^\dagger b_k - \bar{n}_k(T_h)], \tag{S24}$$

$$\mathcal{J}_c := -\hbar\sum_k \omega_k\gamma_k r_k[b_k^\dagger b_k - \bar{n}_k(T_c)]. \tag{S25}$$

This identification is standard in the theory of open quantum systems, and it can be proven [S4] that the time-evolution described by the master equation (S11) fulfills the entropy production inequality:

$$\frac{d\mathcal{S}}{dt} - \frac{\langle\mathcal{J}_h\rangle}{T_h} - \frac{\langle\mathcal{J}_c\rangle}{T_c} \geq 0, \tag{S26}$$

where $\mathcal{S} = -k_B\mathrm{Tr}(\rho\log\rho)$ is the thermodynamical entropy. In the stationary regime, $t\to\infty$, the system approaches some steady state $\lim_{t\to\infty}\rho(t) = \rho_{\mathrm{ss}}$, so that $\frac{d\langle H_S\rangle}{dt} = 0$ and $\frac{d\mathcal{S}_{\mathrm{ss}}}{dt} = 0$, and Eqs. (S20) and (S26) yield

$$\langle\mathcal{J}_h\rangle_{\mathrm{ss}} + \langle\mathcal{J}_c\rangle_{\mathrm{ss}} = 0, \tag{S27}$$

$$\frac{\langle\mathcal{J}_h\rangle_{\mathrm{ss}}}{T_h} + \frac{\langle\mathcal{J}_c\rangle_{\mathrm{ss}}}{T_c} \leq 0. \tag{S28}$$

Since $T_h > T_c$, the above relations impose that $\langle\mathcal{J}_h\rangle_{\mathrm{ss}} = -\langle\mathcal{J}_c\rangle_{\mathrm{ss}} > 0$. This is in agreement with the second law of thermodynamics, in particular in the Clausius formulation as if no work is performed on system, the current goes from hot to cold bath [S5]. Note that if all baths are at the same temperature, $T_h = T_c = T$, once the stationary limit has been reached, the net external current between system and baths becomes obliviously zero.

Coming back to the system of our interest, we can split the current operator corresponding to hot $\mathcal{J}_h$ and cold $\mathcal{J}_c$ baths as a sum of currents operators for individual baths. Specifically, using Eq. (S14),

$$\mathcal{J}_h = -\hbar\sum_k \omega_k\gamma_k s_k[b_k^\dagger b_k - \bar{n}_k(T_h)] = -\hbar\sum_{y=1}^{N}\sum_k \omega_k\gamma_k|\psi_k(1,y)|^2[b_k^\dagger b_k - \bar{n}_k(T_h)] = \sum_{y=1}^{N}\mathcal{J}_h^y, \tag{S29}$$

where $\mathcal{J}_h^y = -\hbar \sum_k \omega_k \gamma_k |\psi_k(1,y)|^2 [b_k^\dagger b_k - \bar{n}_k(T_h)]$ is the current operator accounting for the energy flow between the hot bath at position y and the system. Similarly, $\mathcal{J}_c = \sum_{y=1}^N \mathcal{J}_c^y$, with $\mathcal{J}_c^y = -\hbar \sum_k \omega_k \gamma_k |\psi_k(N,y)|^2 [b_k^\dagger b_k - \bar{n}_k(T_c)]$.

To obtain $\langle \mathcal{J}_h^y \rangle_{\rm ss}$ and $\langle \mathcal{J}_c^y \rangle_{\rm ss}$, we solve the dynamical equation for $\langle b_k^\dagger b_k \rangle$:

$$\frac{d\langle b_k^\dagger b_k \rangle}{dt} = -\gamma[(s_k + r_k)\langle b_k^\dagger b_k \rangle - s_k \bar{n}_k(T_h) - r_k \bar{n}_k(T_c)], \tag{S30}$$

obtaining

$$\langle b_k^\dagger b_k \rangle(t) = e^{-\gamma(s_k+r_k)t} \langle b_k^\dagger b_k \rangle(0) + \frac{[1 - e^{-\gamma(s_k+r_k)t}]}{s_k + r_k} [s_k \bar{n}_k(T_h) + r_k \bar{n}_k(T_c)], \tag{S31}$$

so that

$$\langle b_k^\dagger b_k \rangle_{\rm ss} = \lim_{t\to\infty} \langle b_k^\dagger b_k \rangle(t) = \frac{s_k \bar{n}_k(T_h) + r_k \bar{n}_k(T_c)}{s_k + r_k}. \tag{S32}$$

Therefore, the external currents at the stationary state are

$$\langle \mathcal{J}_h^y \rangle_{\rm ss} = \hbar \sum_k \omega_k \gamma_k r_k |\psi_k(1,y)|^2 \left[\frac{\bar{n}_k(T_h) - \bar{n}_k(T_c)}{s_k + r_k} \right], \tag{S33}$$

$$\langle \mathcal{J}_c^y \rangle_{\rm ss} = \hbar \sum_k \omega_k \gamma_k s_k |\psi_k(N,y)|^2 \left[\frac{\bar{n}_k(T_c) - \bar{n}_k(T_h)}{s_k + r_k} \right]. \tag{S34}$$

In order to derive internal currents we make use of the exact form of the continuity equation and the Davies' theorem [S2]. Specifically, the exact equation for the population at the site (x, y) is given by

$$\begin{aligned} \frac{d\langle a_{x,y}^\dagger a_{x,y} \rangle}{dt} =& \frac{i}{\hbar} \langle [H_S, a_{x,y}^\dagger a_{x,y}] \rangle + \frac{i}{\hbar} \langle [H_{SB}, a_{x,y}^\dagger a_{x,y}] \rangle \\ =& - iJ \langle a_{x+1,y}^\dagger a_{x,y} e^{i\theta_{x,y}^X} - a_{x,y}^\dagger a_{x+1,y} e^{-i\theta_{x,y}^X} \rangle - iJ \langle a_{x-1,y}^\dagger a_{x,y} e^{-i\theta_{x,y}^X} - a_{x,y}^\dagger a_{x-1,y} e^{i\theta_{x,y}^X} \rangle \\ & - iJ \langle a_{x,y+1}^\dagger a_{x,y} e^{i\theta_{x,y}^Y} - a_{x,y}^\dagger a_{x,y+1} e^{-i\theta_{x,y}^Y} \rangle - iJ \langle a_{x,y-1}^\dagger a_{x,y} e^{-i\theta_{x,y}^Y} - a_{x,y}^\dagger a_{x,y-1} e^{i\theta_{x,y}^Y} \rangle \\ & + \frac{i}{\hbar} \langle [H_{SB}, a_{x,y}^\dagger a_{x,y}] \rangle. \end{aligned} \tag{S35}$$

Then, the (internal) current operators are identified as:

$$\mathcal{J}_{(\to x),y} = iJ \big(a_{x,y}^\dagger a_{x-1,y} e^{i\theta_{x,y}^X} - a_{x-1,y}^\dagger a_{x,y} e^{-i\theta_{x,y}^X} \big), \tag{S36}$$

$$\mathcal{J}_{x,(\to y)} = iJ \big(a_{x,y}^\dagger a_{x,y-1} e^{i\theta_{x,y}^Y} - a_{x,y-1}^\dagger a_{x,y} e^{-i\theta_{x,y}^Y} \big), \tag{S37}$$

where the subindex $(\to x)$ is a short notation for $(x - 1 \to x)$. So that $\mathcal{J}_{(\to x),y}$ denotes the operator for the current leaving the site $(x - 1, y)$ and entering in (x, y). Similarly for $(\to y)$.

The term $\frac{i}{\hbar} \langle [H_{SB}, a_{x,y}^\dagger a_{x,y}] \rangle$ in (S35) is not zero only for $x = 1$ and $x = N$, and defines the exact external currents. Of course we cannot compute the exact time derivative $d\langle a_{x,y}^\dagger a_{x,y} \rangle/dt$; our approximation to it is given by the master equation (S11). However, if one tries to define internal currents directly from Eq. (S11), one finds a problem because the approximation introduces fictitious dissipative couplings among all oscillators. Nevertheless, the Davies theorem [S2] asserts that the dissipative part of (S11) is actually a weak-coupling approximation of the term $\mathrm{Tr}_B\,(-i[H_{SB}, \rho])$ [S6]. This suggests that, in a weak coupling regime, it is consistent to take the above exact internal currents operators as internal current operators also in the master equation approximation. In this manner, the terms $\frac{i}{\hbar} \langle [H_{SB}, a_{1,y}^\dagger a_{1,y}] \rangle$ are intended to be described by $\mathcal{J}_h^y$ as defined above.

In a Landau-type gauge taken throughout the main document, we write $\boldsymbol{A} = (-|\boldsymbol{B}|y, 0, 0)$ and internal currents take the form

$$\mathcal{J}_{(\to x),y} := iJ \big(a_{x,y}^\dagger a_{x-1,y} e^{-2\pi\alpha i y} - a_{x-1,y}^\dagger a_{x,y} e^{2\pi\alpha i y} \big), \tag{S38}$$

$$\mathcal{J}_{x,(\to y)} := iJ \big(a_{x,y}^\dagger a_{x,y-1} - a_{x,y-1}^\dagger a_{x,y} \big), \tag{S39}$$

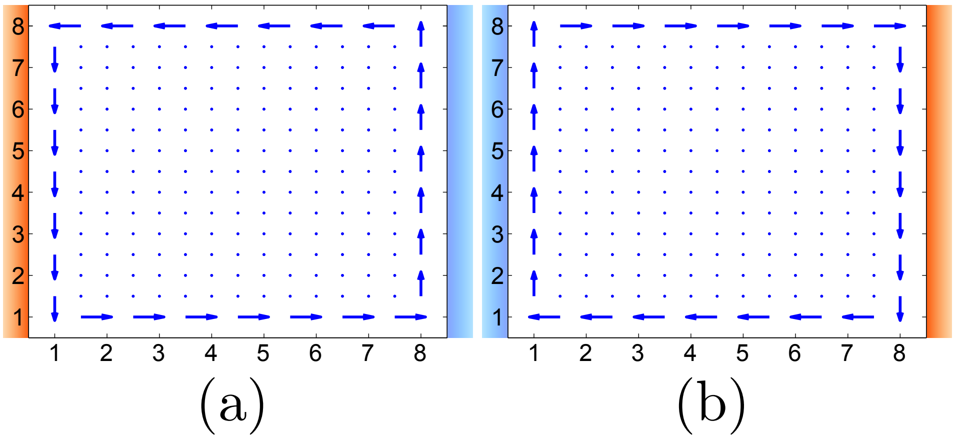

FIG. S2: Current patterns for an out-of-thermal-equilibrium situation. In (a) the lattice is in contact with a bath at $T_h = 2500$ K on the left side and with a bath at $T_c = 1500$ K of the right side. Note the anomalous current on the top edge in opposite direction to the heat flow. The inverted situation along the direction of the temperature gradient is depicted in (b) showing a reversed current.

where α stands for the flux of $\boldsymbol{B}$ per plaquette. These equations, in terms of normal modes, become

$$\mathcal{J}_{(\to x),y} = iJ \sum_{k,k'} u^*_{N(y-1)+x,k} u_{N(y-1)+(x-1),k'} e^{-2\pi\alpha i y} b^\dagger_k b_{k'} + \text{h.c.}, \tag{S40}$$

$$\mathcal{J}_{x,(\to y)} = iJ \sum_{k,k'} u^*_{N(y-1)+x,k} u_{N(y-2)+x,k'} b^\dagger_k b_{k'} + \text{h.c.} \tag{S41}$$

At the stationary limit the steady state is given by Eq. (S19) and we obtain

$$\begin{aligned} \langle \mathcal{J}_{(\to x),y} \rangle &= \frac{J}{2} \text{Im} \sum_k u^*_{N(y-1)+(x-1),k} u_{N(y-1)+x,k} e^{2\pi\alpha i y} \langle b^\dagger_k b_k \rangle \\ &= \frac{J}{2} \sum_k \bar{n}_k (T^{\text{eff}}_k) \text{Im}[u^*_{N(y-1)+(x-1),k} u_{N(y-1)+x,k} e^{2\pi\alpha i y}], \end{aligned} \tag{S42}$$

$$\begin{aligned} \langle \mathcal{J}_{x,(\to y)} \rangle &= \frac{J}{2} \text{Im} \sum_k u^*_{N(y-2)+x,k} u_{N(y-1)+x,k} \langle b^\dagger_k b_k \rangle \\ &= \frac{J}{2} \sum_k \bar{n}_k (T^{\text{eff}}_k) \text{Im}[u^*_{N(y-2)+x,k} u_{N(y-1)+x,k}]. \end{aligned} \tag{S43}$$

Note that these internal currents describe the flux of carriers or quanta per time, we do not aim to associate any specific energy with the current from some site to its adjacent independently of what normal mode is excited in the lattice.

In addition, note also that if $s_k = r_k$, T^{eff}_k is invariant under the exchange $T_h \leftrightarrow T_c$, (because $s_k = r_k$). This, in particular, implies that the internal currents do not change, neither their absolute value nor their sign, under the exchange $T_h \leftrightarrow T_c$. This may be surprising at first sight, but it is due to the fact that a simple exchange of $T_h \leftrightarrow T_c$ can be seen as lattice rotation R_π, because in that case left and right temperature are exchanged and the magnetic field and lattice properties remain invariant. Under such a rotation, it seems natural that currents do not change their value as they are chiral. However, under a reflection along the temperature gradient direction $x \leftrightarrow -x$, the temperatures are exchanged $T_h \leftrightarrow T_c$ but also the magnetic field changes $\boldsymbol{B} \leftrightarrow -\boldsymbol{B}$. In this situation the currents indeed change their sign. This is showed in Fig. S2.

Finally, in Fig. S3 we depict the edge/bulk current ratio as a function of the temperature for the numerical parameters taken throughout the manuscript. We can distinguish the three phases in Fig. 2 of the main text. For low and high temperatures the bulk current is similar to (or higher than) the edge current. However, for a large intermediate range of temperatures the system remains in a phase with high edge current concentration.

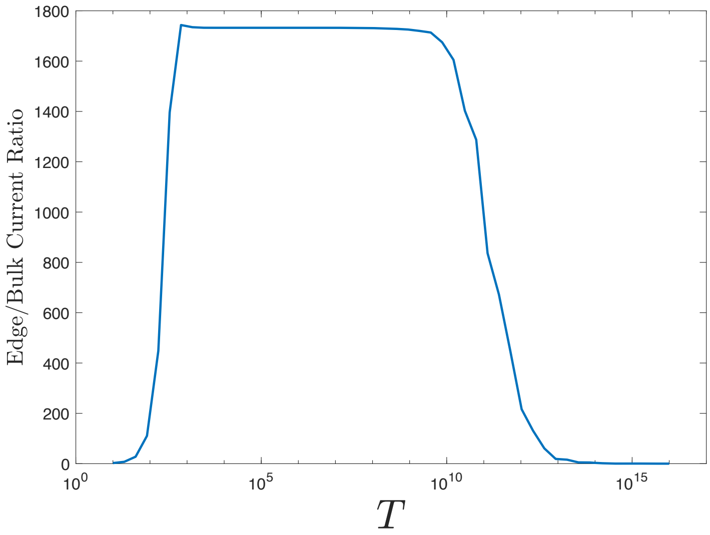

FIG. S3: Ratio between edge and bulk current as a function of the temperature. We have taken a 50×50 array with the same values for sites energies and couplings as in the main text.

MASTER EQUATION IN THE "LOCAL" APPROACH

In the analysis of internal currents it is also common to consider the sometimes refereed to as the "local" approach to the master equation. In this approach the master equation for our system reads

$$\begin{aligned}\frac{d\rho}{dt} = \mathcal{L}(\rho) = -\frac{i}{\hbar}[H_S, \rho] + \gamma \sum_{y=1}^{N} & [\bar{n}(T_h) + 1] \Big(a_{1,y} \rho a_{1,y}^\dagger - \frac{1}{2}\{a_{1,y}^\dagger a_{1,y}, \rho\} \Big) \\ & + \bar{n}(T_h) \Big(a_{1,y}^\dagger \rho a_{1,y} - \frac{1}{2}\{a_{1,y} a_{1,y}^\dagger, \rho\} \Big) \\ & + [\bar{n}(T_c) + 1] \Big(a_{N,y} \rho a_{N,y}^\dagger - \frac{1}{2}\{a_{N,y}^\dagger a_{N,y}, \rho\} \Big) \\ & + \bar{n}(T_c) \Big(a_{N,y}^\dagger \rho a_{N,y} - \frac{1}{2}\{a_{N,y} a_{N,y}^\dagger, \rho\} \Big). \end{aligned} \tag{S44}$$

with $\bar{n}(T) = \{\exp[\hbar\omega_0/(k_B T)] - 1\}^{-1}$.

This master equation corresponds to the introduction of dissipation in a kind of "adiabatic" way; by assuming that the dissipative process is not affected when changing J from 0 to some small parameter (see discussion in [S3, S7]). So, this master equation is expected to provide a good description of the dynamics as long as J is small in comparison with the typical time scale of the system when $J = 0$. In the stationary regime at the limit $t \to \infty$, this equation is not expected to be a good description and it presents some problems from a thermodynamical point of view. Namely,

1. For $T_h = T_c = T$, the state at the long time limit provided by this master equation is not a Gibbs state. Of course, if J is small enough both states are very close [S7], however this does not guaranty a correct thermodynamic description [S8].

2. For $T_h > T_c$, external currents may violate the second law of thermodynamics [S9], accounting for an unphysical heat flow from cold to hot bath.

Yet, for the sake of comparison we have solved this equation (S44): no chiral currents are found, and the pattern is independent of the values of α. This is in agreement with the results reported on [S10] for a lattice with two rows (a ladder) where no chiral current was obtained.

[S1] Hofstadter, D. R. Energy levels and wave functions of Bloch electrons in rational and irrational magnetic fields. *Phys. Rev. B.* **14**, 2239 (1976).

[S2] Davies, E. B. Markovian master equations. *Commun. Math. Phys.* **39**, 91 (1974); Markovian master equations. II. *Math. Ann.* **219**, 147 (1976).

[S3] See for instance: Rivas, A. and Huelga, S. F. *Open Quantum Systems. An Introduction* (Heidelberg: Springer, 2012); Alicki, R. and Lendi, K. *Quantum Dynamical Semigroups and Applications* (Berlin: Springer, 2007).

[S4] Spohn, H. Entropy production for quantum dynamical semigroups. *J. Math. Phys.* **19**, 1227(1978).

[S5] Clausius, R. Ueber eine veränderte Form des zweiten Hauptsatzes der mechanischen Wärmetheorie. *Ann. Phys. (Berlin)* **169**, 481(1854).

[S6] Davies' theorem states $\mathrm{Tr}_B[\exp(-iHt)\rho_S \otimes \rho_B \exp(iHt)] \approx \exp(\mathcal{L}t)\rho_S$. By differentiation $\mathrm{Tr}_B\{-i[H, \exp(-iHt)\rho_S \otimes \rho_B \exp(iHt)]\} \approx \mathcal{L}[\exp(\mathcal{L}t)\rho_S]$. Since $H = H_S + H_B + H_{SB}$, $\mathcal{L}(\cdot) = -i[H_S, \cdot] + \mathcal{D}(\cdot)$ and

$$\mathrm{Tr}_B\{-i[H_S, \exp(-iHt)\rho_S \otimes \rho_B \exp(iHt)]\} = -i[H_S, \mathrm{Tr}_B\{\exp(-iHt)\rho_S \otimes \rho_B \exp(iHt)\}] \approx -i[H_S, \exp(\mathcal{L}t)\rho_S], \quad \text{(S45)}$$

ones concludes $\mathrm{Tr}_B\{-i[H_{SB}, \exp(-iHt)\rho_S \otimes \rho_B \exp(iHt)]\} \approx \mathcal{D}[\exp(\mathcal{L}t)\rho_S]$.

[S7] Rivas, A., Plato, A. D. K., Huelga, S. F., and Plenio, M. B. Markovian master equations: a critical study. *New J. Phys.* **12**, 113032 (2010).

[S8] Čápek, V. Isothermal Maxwell demon as a quantum "sewing machine". *Phys. Rev. E* **57**, 3846 (1998); Novotný, T. Investigation of apparent violation of the second law of thermodynamics in quantum transport studies. *Europhys. Lett.* **59**, 648 (2002).

[S9] Levi, A. and Kosloff, R. The local approach to quantum transport may violate the second law of thermodynamics. *Europhys. Lett.* **107**, 20004 (2014).

[S10] Guo, C. and Poletti, D. Geometry of system-bath coupling and gauge fields in bosonic ladders: Manipulating currents and driving phase transitions. *Phys. Rev. A* **94**, 033610 (2016).
